# Status and influential factors of vitamin D among children aged 0 to 6 years in a Chinese population

**DOI:** 10.1186/s12889-020-08557-0

**Published:** 2020-04-01

**Authors:** Heng Zhang, Zhijuan Li, Yarong Wei, Jinyan Fu, Yaling Feng, Daozhen Chen, Dexiang Xu

**Affiliations:** 1 Department of Toxicology, Anhui Medical University, Hefei, Anhui China; 2 Department of Child Health Care, The Affiliated Wuxi Matemity and Child Health Care Hospital of Nanjing Medical University, Wuxi, Jiangsu China; 3 Wuxi Center for Disease Prevention and Control, Wuxi, Jiangsu China; 4 Department of Obstetrics, The Affiliated Wuxi Matemity and Child Health Care Hospital of Nanjing Medical University, Wuxi, Jiangsu China; 5 Department of Perinatal Health Care, The Affiliated Wuxi Matemity and Child Health Care Hospital of Nanjing Medical University, Wuxi, Jiangsu China; 6 Department of Clinical Laboratory, The Affiliated Wuxi Matemity and Child Health Care Hospital of Nanjing Medical University, Wuxi, Jiangsu China

**Keywords:** Vitamin D, Deficiency, Children age, Season, Air temperature

## Abstract

**Background:**

Vitamin D insufficiency and deficiency in childhood are common. However, the status and influential factors of vitamin D during different ages are not clear. This study aimed to survey vitamin D concentrations in children aged 0 to 6 years and explore its influential factors.

**Methods:**

A total of 6953 children were recruited in Wuxi City of East China from January to December in 2016. Enzyme-linked immunosorbent assay was used to determine the serum concentrations of 25-hydroxyvitamin D [25(OH)D].

**Results:**

The median vitamin D concentrations in the infant group (0–1 years of age) was 69.40 nmol/L, which were higher than that in both the toddlerhood group (1–3 years of age; 62.30 nmol/L) and the preschool group (3–6 years of age; 50.85 nmol/L). In addition, the median vitamin D concentrations were 71.70 nmol/L in summer, which was higher than that in spring (64.25 nmol/L), autumn (62.95 nmol/L) and winter (64.10 nmol/L). However, no difference was observed between genders (*P* = 0.974). Furthermore, the prevalence of vitamin D deficiency (< 50 nmol/L) was 48.1% in the preschool group (3–6 years of age), which was higher than the 21.2% vitamin D deficiency in the toddlerhood group (1–3 years of age) and the 17.9% vitamin D deficiency in the infant group (0–1 years of age). Interestingly, a nonlinear association between 25(OH) D and air temperature was observed.

**Conclusions:**

A high prevalence of vitamin D deficiency was common in a Chinese population of children 0–6 years old, especially in the preschool-aged children. Therefore, we suggested that we should pay more attention to vitamin D supplementation in Chinese young children.

## Background

Vitamin D, including vitamin D2 and D3, was a class of fat-soluble vitamins. Vitamin D2 was most commonly found in vegetable sources and fortified foods [1, 2]. Vitamin D3 can be found in animal-based foods but is mainly synthesized in the skin by a photolytic conversion of cutaneous 7-dehydroxycholesterol by UV sunlight to form previtamin D3 and subsequently vitamin D [3]. 25-hydroxyvitamin D [25(OH)D] is the main form of vitamin D in the body and is also the best indicator to evaluate the concentrations of vitamin D, while 1, 25(OH)2D is the main activated vitamin D compound in the body [4, 5]. Vitamin D has a classic effect on calcium and phosphorus metabolism that impacts bone health. A growing number of studies have linked Vitamin D to extraosseous conditions, including autoimmune diseases, asthma, cardiovascular diseases and infections [6–11].

According to many experts, a 25(OH) D concentrations < 20 ng/mL (50 nmol/L) is defined as deficiency, 20 ~ 29 ng/mL (50~75 nmol/L) as insufficiency, and ≥ 30 ng/mL (≥75 nmol/L) as sufficient [12, 13]. Only 5 to 10% of vitamin D throughout the body is obtained from dietary intake, while more than 90% of vitamin D is derived from cutaneous production [14]. With the increasing air pollution and corresponding decline of outdoor activities, vitamin D deficiency has become increasingly common in humans, especially in children.

Vitamin D insufficiency and deficiency has become a public health problem. A study showed that in America, an insufficiency level of 25(OH) D (50–75 nmol/L) in children aged 6–11 years (73%) was higher than those aged 1–5 years (63%) [15]. Another study showed that the rate of vitamin D deficiency (25(OH) D < 50 nmol/L) was the lowest among infants (5.4%) and was the highest among adolescents (46.4%) [16]. A previous study focusing on vitamin D status in children aged 1–3 years found that the prevalence of vitamin D deficiency was 16.1% among children aged 1~3 years [17]. However, the vitamin D status of children in the preschool period is seldom investigated, especially in Southeast China.

The purpose of this study was to evaluate vitamin D status among children aged 0 to 6 years old in Wuxi City of Southeastern China. Additionally, we also analyzed the influential factors of vitamin D deficiency and insufficiency.

## Methods

### Participants

Our participants were from Health Examination Centers at Wuxi Maternal and Child Health Hospital in Wuxi City, Jiangsu Province, China, from January to December in 2016. The present study obtained ethics approval from the ethics committee of Wuxi Maternal and Child Health Hospital. All participants’ parents or guardians agreed to this study. A total of 6953 children aged 0–6 years old were recruited for this study.

### Collect samples for determination of 25 (OH) D

Blood sample was collected by using a finger stick for each participant. Within 10 min after collection, the blood sample was centrifuged at 3500 rpm for 15 min. All sera samples were stored at − 80 °C until analysis. The serum 25(OH) D concentrations were determined by enzyme-linked immunosorbent assay following the manufacturer’s instructions (IDS Ltd., Boldon Colliery, Tyne & Wear, UK) [17]. The inter-assay and intra-assay coefficients of variation were < 10%.

### Statistical analysis

The distribution of serum 25(OH) D concentrations among the children was positively skewed. Therefore, percentiles and medians were used to describe the serum 25(OH) D concentrations. The season during which each serum sample was classified by the following standard: spring (March to May); summer (June to August); fall (September to November); winter (December to February) [17]. We conducted analyses of serum 25(OH) D concentrations stratified by age group, season and gender. The children were divided into age groups according to clinical criteria [18] as follows: infant group was defined as the stage of life from birth to ≤1 years old (0–1 years of age); toddlerhood group was defined as the age group between > 1 and ≤ 3 years old (1–3 years of age); preschool group was defined as the age group between > 3 and ≤ 6 years old (3–6 years of age).

A logistic regression model was used to examine odds ratios (*ORs*) and 95% confidence intervals (95% *CIs*) in the relationship between vitamin D deficiency and age group. We performed all statistical analyses with SPSS (version 16.0). A two-tailed *P*-value less than 0.05 was considered statistically significant. To further evaluate the possible nonlinear relationships between 25(OH) D with air temperature, locally weighted regression was applied by using BStudio.

## Results

### Status of vitamin D in children aged 0–6 years old

A population of 6953 children (3749 boys and 3204 girls) aged 0 to 6 years was recruited in this study. The median (*P*_5_-*P*_95_) concentrations of serum 25(OH) D were 65.40 (34.00–102.70 nmol/L) in the total population (Table 1).

**Table 1:** Comparison of serum 25(OH) D levels in 6953 young children stratified by age, season or gender

Variables	Number	Median (IQR)	*P*_5_–*P*_95_	*P* value
Total population	6953	65.40 (51.65–81.50)	34.00–102.70	
Age group				
Infant group	4603	69.40 (55.00–85.30)	34.50–106.70	**< 0.001**
Toddlerhood group	1546	62.30 (51.70–77.20)	38.60–94.87	
Preschool group	804	50.85 (40.65–62.70)	30.60–80.70	
Season				
Spring	2150	64.25 (50.30–80.50)	33.10–100.80	**< 0.001**
Summer	1727	71.70 (55.70–87.30)	37.30–107.60	
Autumn	1392	62.95 (49.42–78.80)	34.36–104.70	
Winter	1684	64.10 (51.10–78.40)	33.50–100.00	
Gender				
Boy	3749	65.60 (51.30–81.50)	34.25–102.30	0.974
Girl	3204	65.20 (52.00–81.57)	33.90–103.95	

Abbreviation: *IQR*, Interquartile range

The median concentrations of 25(OH) D in the infant group (0–1 years of age) were 69.40 nmol/L, which were higher than that in toddlerhood group (1–3 years of age; 62.30 nmol/L) and that in preschool group (3–6 years of age; 50.85 nmol/L) (Table 1). Meanwhile, the median concentrations of vitamin D were 71.70 nmol/L in summer, which was higher than that in spring (64.25 nmol/L), autumn (62.95 nmol/L) and winter (64.10 nmol/L). However, no difference in median concentrations of vitamin D between the genders were observed (*P* = 0.974) (Table 1).

In addition, there was a high prevalence of vitamin D deficiency (22.1%) and insufficiency (43.2%) among children aged 0–6 years old in the Chinese population (Table 2). The prevalence of vitamin D deficiency was 17.9% in the infant group (0–1 years of age), which was lower than that in the toddlerhood group (1–3 years of age; 21.2%) and that in the preschool group (3–6 years of age; 48.1%) (Table 2).

**Table 2:** The groups of vitamin D concentrations stratified by age, season and gender

Variables	Vitamin D groups			*P* value
	Deficiency (< 50 nmol/L); %	Insufficient (50–74.9 nmol/L); %	Sufficient (≥ 75 nmol/L); %	
Total population	22.1	43.2	34.7	
Age group				
Infant group	17.9	40.8	41.3	**< 0.001**
Toddlerhood group	21.2	50.6	28.2	
Preschool group	48.1	42.2	9.7	
Season				
Spring	24.4	42.7	32.9	**< 0.001**
Summer	15.8	39.1	45.1	
Autumn	26.0	43.4	30.6	
Winter	22.4	47.7	29.9	
Gender				
Boy	22.7	42.2	35.1	0.167
Girl	21.4	44.3	34.3	

### Factors influencing vitamin D deficiency

The logistic regression analysis revealed that vitamin D deficiency and insufficiency were strongly associated with children age (Table 3). Compared to serum 25(OH) D ≥ 75 nmol/L, the *OR* for vitamin D deficiency (< 50 nmol/L) in the toddlerhood group (1–3 years of age) and the preschool group (3–6 years of age) were 1.73 (95% *CI*: 1.47, 2.04) and 11.5 (95% *CI*: 8.87, 14.81), respectively. The *ORs* for vitamin D insufficiency (50–74.9 nmol/L) in the toddlerhood group (1–3 years of age) and preschool group (3–6 years of age) were 1.82 (95% *CI*: 1.59, 2.08) and 4.40 (95% *CI*: 3.41, 5.67), respectively. After adjustment for confounding factors, the association remained (Table 3).

**Table 3:** Odds ratios (95% CIs) of vitamin D deficiency and vitamin D insufficient associated with age group

	Vitamin D deficiency (< 50 nmol/L) ^a^		Vitamin D insufficient (50–74.9 nmol/L) ^a^	
	Univariate OR (95% CI)	Adjusted ^b^ OR (95% CI)	Univariate OR (95% CI)	Adjusted ^b^ OR (95% CI)
Age group				
Infant group	1.00	1.00	1.00	1.00
Toddlerhood group	1.73 (1.47, 2.04)^***^	1.76 (1.49, 2.08)^***^	1.82 (1.59, 2.08)^***^	1.83 (1.60. 2.10)^***^
Preschool group	11.5 (8.87, 14.81)^***^	12.22 (9.42, 15.84)^***^	4.40 (3.41, 5.67)^***^	4.60 (3.56, 5.94)^***^

^a^ The reference group was serum 25(OH) D ≥ 75 nmol/L.

^b^ Adjusted for season and genders

^***^P < 0.001

**Fig. 1:**
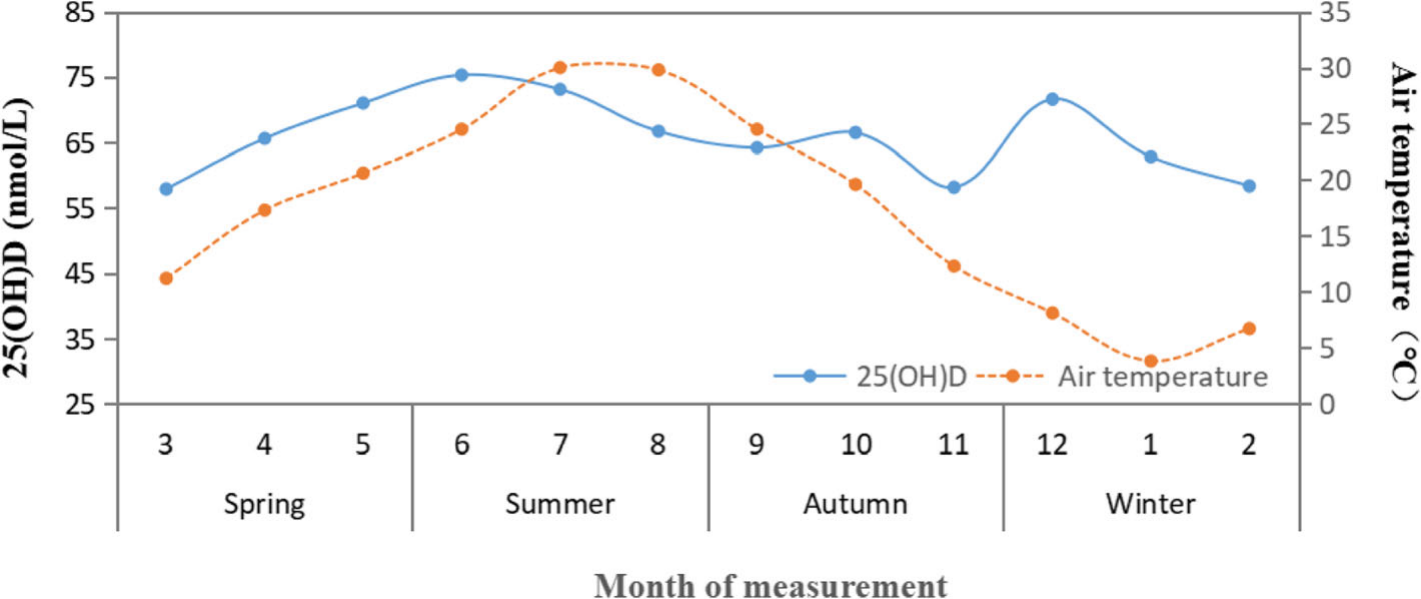
Trends in 25(OH) D and average monthly air temperature variation with season. From January to December 2016, the 25(OH) D levels and average monthly air temperature values showed variation and the shapes of the curves

**Fig. 2:**
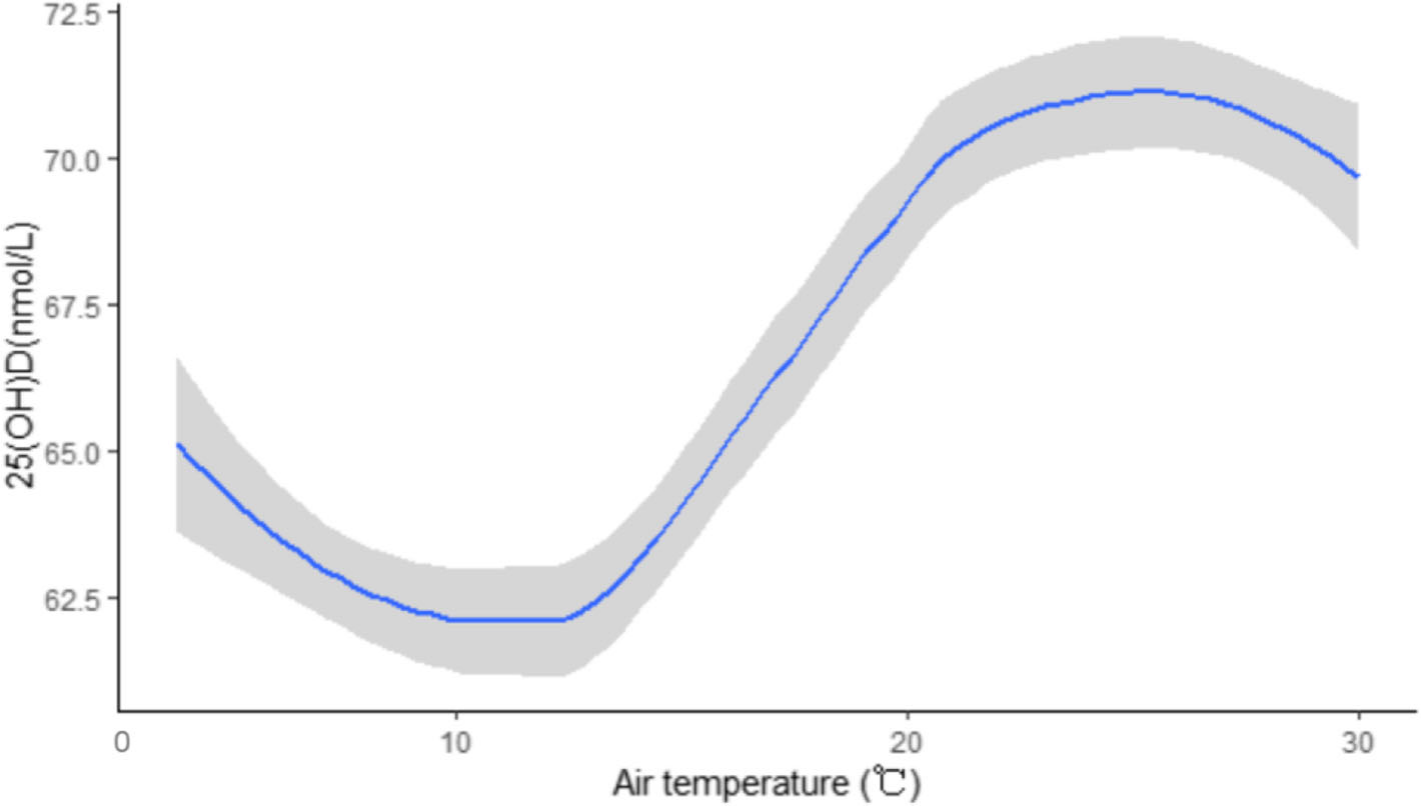
The possible non-linear association between 25(OH) D and air temperature. With increasing air temperatures, the 25(OH) D levels decreased then increased to a high level and declined slightly again

The trends in 25(OH) D, together with the average monthly air temperature variation, were shown in Fig. 1. The results showed that the changes in vitamin D concentrations were not consistent with the changes of air temperature. In Fig. 2, the nonlinear curve for the association between 25(OH) D and air temperature was shown. With increasing air temperatures, the 25(OH) D concentrations first decreased at air temperature in the range of 0–10 °C, then increased to a high level at air temperature in the range of 10–24 °C, and then again declined slightly at air temperature over 24 °C.

## Discussion

Vitamin D deficiency and insufficiency are global problems [19–21]. Our study found that a high prevalence of vitamin D deficiency and insufficiency among children aged 0–6 years old in a Chinese population, especially in the preschool-aged children.

As for serum 25(OH) D concentration, only 34.7% of the children were sufficient, 43.2% were insufficient, and 22.1% were deficient in the total population. The prevalence of vitamin D deficiency (< 50 nmol/L) increased by age, with 17.9, 21.2 and 48.1% in the infants (0–1 years of age), toddlers (1–3 years of age) and preschoolers (3–6 years of age) groups, respectively. Consistent with our findings, the prevalence of vitamin D deficiency increased by age in American children, with a deficiency prevalence of 14, 20, and 28.8% in children aged 2–5 years, 6–11 years and adolescents, respectively [15, 22].

In addition, a dramatic seasonal variation of vitamin D deficiency and insufficiency was observed in our present study; the concentrations were very low in autumn, increased gradually in spring and winter, and reached a peak in summer. This is consistent with the results reported in other studies [16, 23–26].

There were many reasons for vitamin deficiency and insufficiency. Vitamin D status in children age has been greatly investigated all over the world. Our finding demonstrated that the prevalence of vitamin D deficiency (< 50 nmol/L) among children aged 0–6 years old in a Chinese population increased by age. This may be due to the Pediatrics Branch of Chinese Medical Association recommends that all children receive no less than 400 IU/day of vitamin D from 2 weeks to 2 years after birth [27]. However, when the children reach ages older than 2 years, the origin of Vitamin D is mainly from outdoor activities. Studies have shown that children have lower concentrations of vitamin D due to less outdoor activities [28–30].

In addition, for most people, the majority (80–90%) of the 25(OH) D in the circulation is produced by the skin’s 7-dehydrocholesterol by ultraviolet B radiation [31]. Therefore, sunlight exposure maybe was the primary factor affecting the vitamin D status in children. Also, the circulating 25(OH) D is regularly influenced by season [32, 33]. The seasonal variation in vitamin D status correlated well with the seasonal variation in the intensity of solar UVB light [34, 35]. Additionally, the condition of UVB on skin radiation determines the effect of temperature on vitamin D status [36, 37]. One study reported that the association between 25(OH) D concentrations and air temperature and revealed that the 25(OH) D concentrations were consistent with the changes in air temperature [37]. Interestingly, our research found a nonlinear curve for the association between 25(OH) D concentrations and air temperature in the pediatric population. Some children may be taken vitamin D supplements due to less outdoor activities during the transition from winter to spring. However, vitamin D supplementation may decline as air temperatures rise, which was a probable reason for the decreasing vitamin D concentrations in the temperature range of 0–10 °C. Then the 25(OH) D concentrations increased to a high level when the air temperature increased from 10 °C to 24 °C. It was probable that the children had more outdoor activities in this comparatively more comfortable temperature range. However, when the air temperature exceeded 24 °C, the 25(OH) D levels again declined slightly with rising air temperatures. Children spent less time outdoors due to high air temperatures [38], which is a possible reason for the decreasing vitamin D levels.

There were some limitations of our study worth mention. The socioeconomic status of the participants has not been provided in this study. Additionally, our study did not collect information regarding the children’s dietary intake, vitamin D supplements, body mass index, time of physical activities or amount of sunlight exposure. These factors might affect the vitamin D status of young children.

## Conclusions

A high prevalence of vitamin D deficiency was common in this population of Chinese children between 0 and 6 years old, especially in the preschool-aged children. Taking into consideration the lack of vitamin D in children and adolescents, the American Academy of Pediatrics has issued a new recommendation that all children receive 400 IU/day of vitamin D daily from the first day of life to adolescence [39]. Therefore, we suggested that we should pay more attention to vitamin D supplementation in Chinese young children.

## Data Availability

The study data can be obtained from the corresponding author on reasonable request.

## Abbreviations

25(OH)D: 25-hydroxyvitamin D
ORs: Odds ratios
CIs: Confidence intervals
